# Receptor-Heteromer Investigation Technology and its application using BRET

**DOI:** 10.3389/fendo.2012.00101

**Published:** 2012-08-22

**Authors:** Elizabeth K. M. Johnstone, Kevin D. G. Pfleger

**Affiliations:** ^1^ Laboratory for Molecular Endocrinology – GPCRs, Western Australian Institute for Medical Research and Centre for Medical Research, The University of Western AustraliaPerth, WA, Australia; ^2^ Dimerix Bioscience Pty LtdPerth, WA, Australia

**Keywords:** Receptor-HIT, GPCR-HIT, GPCR, RTK, heteromer, BRET, bioluminescence resonance energy transfer

## Abstract

Receptor heteromerization has the potential to alter every facet of receptor functioning, leading to new pharmacological profiles with increased signaling diversity and regulation from that of the monomeric receptor, or indeed receptor homomer. An understanding of the molecular consequences of receptor heteromerization will provide new insights into the physiology and pathology mediated by receptors, expanding the possibilities for pharmacological discovery. Particularly advantageous approaches to investigate novel heteromer pharmacology utilize cell-based assay technologies that assess ligand-dependent functional responses specific to the receptor heteromer. Importantly, this allows for differentiation of heteromer-specific pharmacology from pharmacology associated with the co-expressed receptor monomers and homomers. The Receptor-Heteromer Investigation Technology (Receptor-HIT) successfully employs a proximity-based reporter system, such as bioluminescence resonance energy transfer (BRET), in a configuration that enables determination of such heteromer-specific pharmacology. Therefore, Receptor-HIT provides a simple, robust and versatile approach for investigating the elusive “biochemical fingerprint” of receptor heteromers.

## INTRODUCTION

There are many types of membrane receptors that can be broadly classified into three families based on distinct mechanisms of signal transduction, namely G protein-coupled receptors (GPCRs), receptor tyrosine kinases (RTKs), and ionotropic receptors, which are channels that directly allow flux of ions upon activation. Additionally, there are intracellular receptors such as those binding steroids. It is well established that many of these receptors exist as oligomeric species consisting of two or more receptor subunits (Neubig et al., 2003). In many cases, dimerization or oligomerization is required for the formation of a functional receptor unit. These receptors are known as “homomeric receptors” if the constituents are the same and “heteromeric receptors” if the constituents differ (Ferré, 2009). RTKs are the archetypal homomeric and heteromeric receptors, as they require homo- or heteromerization for activation and signaling (Lemmon and Schlessinger, 2010). For GPCRs, classic examples of heteromeric receptors are the GABA_B_ receptor (GABA_B_R1-GABA_B_R2; Jones et al., 1998; Kaupmann et al., 1998; White et al., 1998) and taste receptors (T1R-T2R and T2R-T3R; Nelson et al., 2001, 2002; Li et al., 2002). In contrast, “receptor homomers” and “receptor heteromers” are macromolecular complexes that include two or more functional receptor units (identical or different, respectively) and display pharmacology that is distinct from that of their component receptors (Ferré, 2009). The concepts of GPCR homomerization and heteromerization have been described for 30 years (Fuxe et al., 2010), but have only recently become widely accepted. Furthermore, it is now clear that an array of receptor homomers and heteromers from all classes of membrane receptors exist (Liu et al., 2000, 2006; Maudsley et al., 2000; Lee et al., 2002; Nair and Sealfon, 2003; Olivares-Reyes et al., 2005; Watt et al., 2009). As both receptor homomers and receptor heteromers have the potential to attain a unique pharmacological profile, their existence adds another level of complexity to cell signaling systems. Of the two classes, receptor heteromers have been the major focus of research interest due to the numerous potential receptor combinations, as well as the difficulty in separating the pharmacology of a monomer from its homomer. The unique pharmacology associated with receptor heteromers has been termed its “biochemical fingerprint” (Ferré, 2009) and provides a mechanism for achieving greater signaling diversity and specificity. Receptor heteromers are therefore viewed as a new class of drug target, providing the opportunity for designing heteromer-specific/-biased drugs with improved selectivity and reduced side effects (Mustafa et al., 2010).

 Investigating the pharmacological properties of receptor heteromers can be a particularly difficult process as the heteromer-specific pharmacology needs to be differentiated from pharmacology of associated monomers/homomers. Due to difficulties in investigating heteromers in native tissue, heterologous expression systems currently provide the major method to study heteromers. The first step is the identification of a heteromer, and subsequent characterization of its biochemical fingerprint. In time, this biochemical fingerprint will ideally be used to demonstrate the presence of the heteromer in native tissue. To achieve this end, it is critical that the initial cell-based assays employed are able to robustly differentiate heteromer-specific pharmacology from that of the component receptors.

## RECEPTOR-HETEROMER INVESTIGATION TECHNOLOGY

**FIGURE 1:**
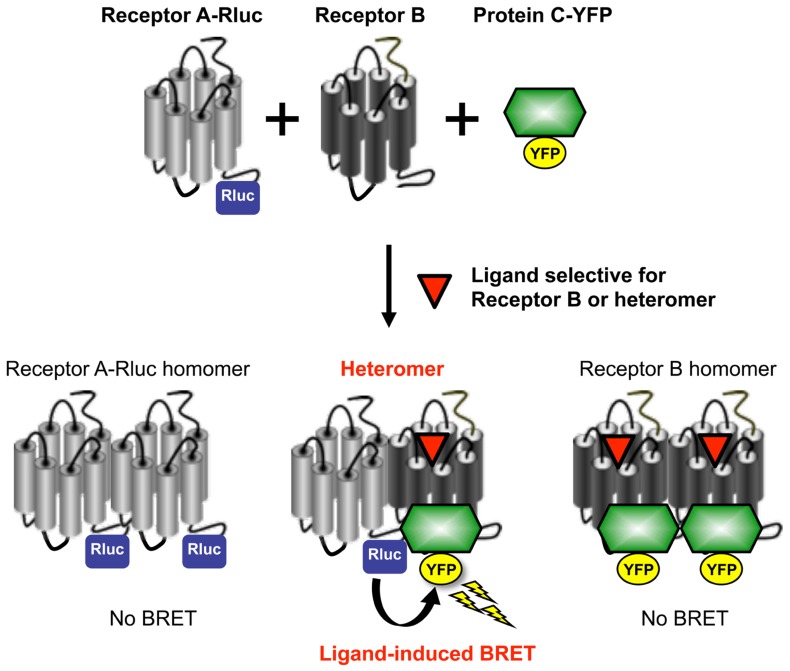
**Utilization of Receptor-HIT on the BRET platform to investigate receptor heteromerization.** BRET is a biophysical phenomenon involving the non-radiative transfer of energy from a donor enzyme to an acceptor fluorophore. The donor enzyme is a variant of *Renilla* luciferase (Rluc), and energy emission results from the oxidation of its substrate coelenterazine h to coelenteramide (Pfleger and Eidne, 2006; Pfleger et al., 2006). If the acceptor fluorophore (such as yellow fluorescent protein; YFP) is correctly orientated and within 10 nm, it will be excited by the energy transferred from the donor resulting in the emission of light at a characteristic wavelength (Dacres et al., 2010). BRET is used to study protein-protein interactions through tagging one of the proteins of interest with the donor enzyme and the other protein with the acceptor fluorophore (Pfleger and Eidne, 2006). If the two proteins are in close proximity, the energy generated by the donor enzyme will be transferred to the acceptor fluorophore. The resulting BRET signal provides evidence for the two fusion proteins being in the same complex. In contrast to BRET between Rluc and YFP fused to each receptor, a specific receptor heteromer can be monitored by ligand-induced BRET due to proximity of a tagged receptor and a tagged intracellular protein used as a reporter partner (such as β-arrestin or G protein). In this system, energy transfer is measured between Receptor A-Rluc and a Protein C-YFP that interacts with the heteromer complex after selective activation of Receptor B or the heteromer itself. In addition to the induction of BRET by the Receptor B or heteromer-selective ligand providing evidence for Receptor AB heteromerization, it also identifies a biological function of the heteromer. Reprinted from Ayoub and Pfleger (2010). Copyright © 2010, with permission from Elsevier.

 A novel technique recently developed to enable identification and pharmacological profiling of heteromers is the Receptor-Heteromer Investigation Technology (Receptor-HIT). This provides information on ligand-dependent functional responses specific to the heteromer. Receptor-HIT uses a proximity-based reporter system comprising four elements, three of which are: labeled Receptor A, untagged Receptor B, and a labeled interacting Protein C that is recruited to the heteromer in a ligand-dependent manner (See et al., 2011). This configuration is illustrated in **Figure 1** using bioluminescence resonance energy transfer (BRET), however, the approach can be applied using a variety of reporter systems including fluorescence resonance energy transfer (FRET), bimolecular fluorescence complementation (BiFC), bimolecular luminescence complementation (BiLC), enzyme fragment complementation (EFC), and the protease-cleaved transcription factor assay system known as Tango^TM^ (Mustafa et al., 2010; Mustafa and Pfleger, 2011). Co-expression of the aforementioned elements in cells enables the signal between the label of choice on Receptor A and complementary label on Protein C to be monitored. The fourth element in the system is a ligand that, upon binding to untagged Receptor B or the heteromer, selectively modulates the recruitment of Protein C to Receptor B and/or the heteromer (See et al., 2011; Mustafa et al., 2012). Receptor-HIT is unsuitable for investigating homomers due to this receptor-selectivity requirement, but heteromers of closely related receptor subtypes where a selective agonist may be unavailable can still be assessed. This issue is overcome by additional use of an antagonist selective for Receptor A, thereby meaning that Receptor B and/or the heteromer are still activated selectively. Alternatively, it is possible to use a non-selective ligand if it does not modulate recruitment of Protein C to Receptor A in the absence of Receptor B (Porrello et al., 2011). Whichever approach is used, generation of a signal upon application of the ligand indicates that Protein C has been recruited to the heteromer, thereby bringing the label on Receptor A into close proximity with the label on Protein C. The signal obtained is not only indicative of the receptors being in a heteromeric complex, it also reveals an aspect of the heteromer’s pharmacology through generation of ligand-dependent functional responses.

Receptor-HIT is an excellent assay for identifying and profiling heteromers as signals do not result from the homomeric or monomeric receptor populations (See et al., 2011). The ligand-dependent nature of the signal also enables screening, identification, and profiling of compounds exhibiting heteromer-specific or biased signaling (Mustafa and Pfleger, 2011; Mustafa et al., 2012).

 The Receptor-HIT assay has largely been published with respect to GPCRs in the form of the GPCR-Heteromer Identification Technology (GPCR-HIT; Ayoub and Pfleger, 2010; Mustafa et al., 2010, 2012; Mustafa and Pfleger, 2011; Porrello et al., 2011; See et al., 2011), however it can also be applied to other receptors, including RTKs (Pfleger, 2011; Story et al., 2011), ionotropic receptors and steroid receptors. Consequently, there is also an extensive number of interacting partners that can be used. For example, GPCR-HIT studies can utilize G proteins or β-arrestins, whereas we have found Grb2 to be particularly amenable to Receptor-HIT assays investigating RTKs (Pfleger, 2011; Story et al., 2011).

BRET is our preferred platform for Receptor-HIT (**Figure 1**) because it can monitor protein proximity in live cells in real time at 37°C without the need for cell lysis, the assay does not rely upon proteins refolding in a complementation event to produce a readout, and no alteration of receptor function is required (Mustafa et al., 2010). The traditional configuration for studying receptor heteromers using BRET involves tagging one receptor with the Rluc enzyme, while the second receptor is tagged with the acceptor fluorophore. A particular limitation to this approach is that overcrowding of receptors in the endoplasmic reticulum or degradative compartments can lead to non-specific “bystander BRET” (Pfleger and Eidne, 2006). This is commonly addressed by employing BRET saturation assays (Mercier et al., 2002), however these are rather laborious. The ligand dependency of Receptor-HIT addresses this issue as it requires Receptor B or the heteromer to be capable of binding ligand (**Figure 1**), either because it is sufficiently mature and/or because it is appropriately localized to provide the ligand access for binding. Furthermore, although providing evidence of proximity of the two receptors, no functional information about the heteromer is revealed by saturation assays (Mustafa et al., 2012). In contrast, the use of an interacting protein also enables functional responses to be assessed, with the potential to uncover novel heteromer-specific pharmacology (Mustafa et al., 2012).

 While there are advantages to using BRET as outlined above, there are also advantages to using other platforms in certain situations. For example, although EFC is not a real-time assay and requires cell lysis for signal detection, it is probably capable of achieving higher levels of screening throughput than BRET. Furthermore, assay systems like FRET and BiFC are more amenable to assessing subcellular localization if combined with confocal microscopy. However, because FRET uses a fluorophore as donor, there are issues arising from the need for external excitation. These include autofluorescence, photobleaching, cell damage, and direct acceptor excitation. Some of these issues can be addressed using time-resolved FRET (TR-FRET; Cottet et al., 2012). BiFC enables specific visualization of complemented fluorophores, and therefore the fused proteins of interest, but this is not a real-time assay due to a time delay while refolding occurs and once complemented, the proteins remain associated (Porrello et al., 2011). BRET is very sensitive to distance and relative donor–acceptor orientation. This is advantageous in terms of proximity specificity, however, it means that receptors could potentially form a heteromer without this being detected by BRET, and a lack of signal should be interpreted with caution (Pfleger et al., 2006). Other platforms may have a lower false-negative rate than BRET, however, the potential for higher false-positives may then need to be considered.

## APPLICATION OF RECEPTOR-HIT USING BRET

 Receptor-HIT has been used effectively on the BRET platform to investigate multiple established and novel heteromers. The CCR2-CCR5 and CCR2-CXCR4 heteromers that have been described by a number of studies (Mellado et al., 2001; Rodríguez-Frade et al., 2004; El-Asmar et al., 2005; Percherancier et al., 2005; Springael et al., 2006; Sohy et al., 2007, 2009) have recently been profiled in terms of dose–response curves, kinetics and *Z*′ data using GPCR-HIT (See et al., 2011). Of particular note were the findings with the combination of CXCR4/Rluc8, β-arrestin2/Venus and CCR2. Treatment with CXCL12 (CXCR4 agonist) resulted in a relatively transient BRET signal that returned to baseline before 40 min, whereas addition of CCL2 (CCR2 agonist) resulted in a more prolonged BRET kinetic profile, indicative of CCR2 forming a complex with CXCR4. Intriguingly, treatment with a combination of CXCL12 and CCL2 resulted in a prolonged and substantially higher BRET signal than observed with either agonist alone. Possible explanations for this include β-arrestin2 recruitment being facilitated by both types of receptor complex being in active receptor conformations, or proximity of the donor and acceptor being sufficiently close to enable detection of changes in donor–acceptor distance and/or relative orientation. Either way, this observation provides good evidence for specific reporting of β-arrestin2 recruitment to the heteromer complex (See et al., 2011).

When generating dose–response curves with Receptor-HIT data, the Hill slope has been seen to alter for particular combinations depending upon whether the tagged or untagged receptor is activated, consistent with stabilization of distinct complex conformations with the different ligands. For example, with the CCR5/Rluc8, β-arrestin2/Venus and CCR2 combination, the dose–response curve with CCL2 was significantly steeper than with CCL4 (CCR5 agonist; See et al., 2011). As discussed previously, the reason for this difference is currently unclear, however, as the protein expression profile is identical in both cases and the only difference is the agonist treatment, this observation may help to shed light on the mechanism of GPCR heteromerization and/or allosterism across the complex in the future (See et al., 2011).

Receptor-HIT (in the form of GPCR-HIT) has also been used to investigate the heteromer between the angiotensin II (AngII) type 1 receptor (AT_1_R) and the AngII type 2 receptor (AT_2_R; Porrello et al., 2011). A number of studies have shown that the AT_2_R does not couple to arrestins and does not internalize following treatment with AngII (Pucell et al., 1991; Hunyady et al., 1994; Turu et al., 2006). Our BRET data indicating a lack of β-arrestin2/Rluc8 recruitment to AT_2_R/Venus are also consistent with these findings (Porrello et al., 2011). Therefore, upon co-expression of untagged AT_1_R with β-arrestin2/Rluc8 and AT_2_R/Venus, even though AngII can bind to both receptors, the ligand is still selective in terms of recruiting β-arrestin2 to only the untagged receptor. Therefore, the observation that a ligand-induced BRET signal results upon addition of AngII is indicative of AT_1_R-AT_2_R heteromerization (Porrello et al., 2011).

**FIGURE 2:**
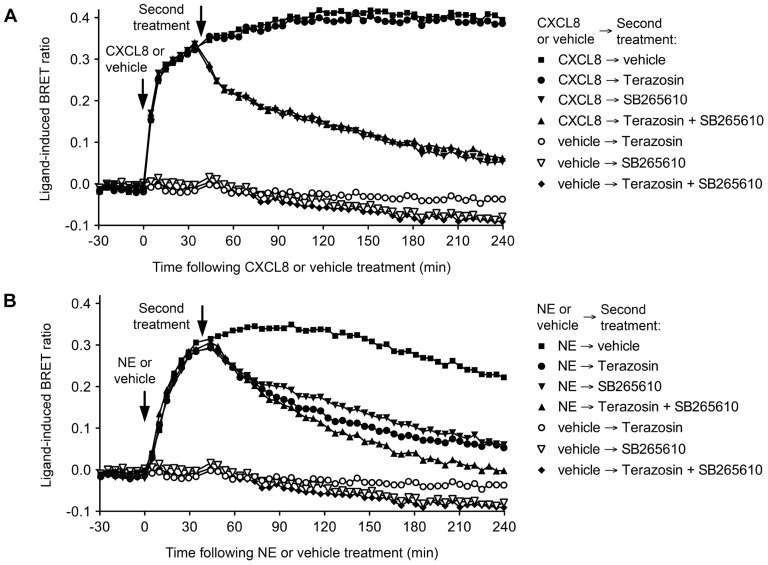
**Use of Terazosin (α_1_AR antagonist) and SB265610 (CXCR2 inverse agonist) to interrogate β-arrestin2 recruitment to the α_1A_AR-CXCR2 heteromer.** Extended BRET kinetic profiles were generated for the CXCR2/Rluc8, β-arrestin2/Venus and α_1A_AR combination in HEK293FT cells by treating with CXCL8 or vehicle **(A)** or NE or vehicle **(B)** ~30 min before a second treatment with vehicle, 10 μM Terazosin, and/or 10 μM SB265610. Data are representative of three independent experiments. This research was originally published in Mustafa et al. (2012). Copyright © 2012 the American Society for Biochemistry and Molecular Biology.

Receptor-HIT on the BRET platform has recently been used to characterize the novel heteromer between the α_1A_-adrenoceptor (α_1A_AR) and the CXC chemokine receptor 2 (CXCR2) that may play a role in prostate stroma (Mustafa et al., 2012). The Receptor-HIT studies showed that the heteromer recruits β-arrestin2 in a norepinephrine (NE)-dependent manner that can be blocked by both the α_1_AR antagonist Terazosin and the CXCR2-specific allosteric inverse agonist SB265610 (**Figure 2**). This is despite the very weak β-arrestin2 interaction with α_1A_AR monomers/homomers in transfected human embryonic kidney 293 cells (Stanasila et al., 2008), but consistent with the observation of α_1A_AR recruiting β-arrestin2 in prostate stroma (Hennenberg et al., 2011). The specificity of this change in α_1A_AR pharmacology with co-expression of CXCR2 was demonstrated by the lack of effect upon co-expression of CC chemokine receptor 2, vasopressin receptor 2 (V2R), or orexin receptor 1 (Mustafa et al., 2012).

The ligand-dependent nature of Receptor-HIT enables it to report on, albeit without differentiating between, constitutive and dynamic heteromers (Mustafa and Pfleger, 2011). The α_1A_AR-CXCR2 complex is an example of a constitutive heteromer that exhibits novel pharmacology revealed by the ligand dependency of Receptor-HIT (Mustafa et al., 2012). Indeed, BRET saturation assays indicated strong specific BRET signals between α_1A_AR/Rluc8 and both CXCR2/Venus and V2R/Venus, however, the functional change in α_1A_AR pharmacology revealed by GPCR-HIT was only observed with CXCR2 and not with V2R. This indicates that proximity between GPCRs does not necessarily result in a functional effect of one receptor on another, and further demonstrates the ability of Receptor-HIT to unmask specific heteromer functionality (Mustafa et al., 2012).

Receptor-HIT is also able to investigate the functionality of apparent dynamic receptor interactions, even though it is unable to determine the dynamics *per se*. This is seen for the heteromer between the glucagon-like peptide-1 receptor (GLP-1R) and the gastric inhibitory polypeptide receptor (GIPR; Schelshorn et al., 2012). Using the combination of GLP-1R-Rluc8 and YPet-β-arrestin2 in the absence and presence of GIPR, dose–response data were generated indicating that expression of GIPR partially inhibited GLP-1-induced recruitment of YPet-β-arrestin2 proximal to GLP-1R-Rluc8. This inhibition was overcome by co-treatment with GIP (Schelshorn et al., 2012). The authors of this study suggested a model to explain their GPCR-HIT data, whereby formation of the heteromer occurs as a consequence of low affinity binding of GLP-1 to the GIPR in addition to the GLP-1R. The heteromer is proposed to recruit β-arrestin2 less well in comparison with the GLP-1R monomer/homomer. Co-treatment with GIP is therefore suggested to compete off the GLP-1 from GIPR, resulting in the heteromer being dissolved and allowing improved GLP-1-induced recruitment of β-arrestin2 to GLP-1R (Schelshorn et al., 2012).

Receptor heteromers are complexes with unique pharmacology that are likely to be expressed in a distinct tissue-specific manner. This makes them exciting new prospects as drug targets, with the goal of developing drugs with improved selectivity and reduced side effects. Indeed, the concept of biased signaling is now well established and applies as readily to receptor heteromers as it does to monomers/homomers (Mustafa et al., 2010). Therefore, heteromerization provides enormous opportunities for identifying ligands with heteromer-selective and/or heteromer-biased pharmacology, such as that observed with Labetalol acting at the α_1A_AR-CXCR2 heteromer (Mustafa et al., 2012).

Screening is an essential step in the identification of lead compounds, and consequently there is a need to develop heteromer assays that are compatible with this process. An assay’s suitability for screening can be gauged by its *Z*′ value; *Z*′ values >0.5 indicate assays that are highly suitable (Zhang et al., 1999). The potential of Receptor-HIT as a screening assay has been demonstrated with the CCR2-CCR5 and CCR2-CXCR4 chemokine receptor heteromers for which a *Z*′ value of 0.68 was generated with both combinations (See et al., 2011). The *Z*′ value for the α_1A_AR-CXCR2 heteromer (Mustafa et al., 2012) was 0.87 (See and Pfleger, unpublished observations). Unlike profiling, screening with Receptor-HIT is a two-step process. Using BRET for example, compounds are firstly screened with the Receptor-HIT configuration of Receptor A-Rluc, Receptor B and Protein C-YFP (**Figure 1**). This may generate hits for ligands that bind to Receptor A, Receptor B (when in a heteromer) or the heteromer specifically. These hits are then rescreened in parallel with the configurations Receptor A-Rluc with Protein C-YFP, and Receptor B-Rluc with Protein C-YFP. This will then enable differentiation between ligands that bind to Receptor A directly, ligands that bind to Receptor B directly, and those that are heteromer-selective (revealed by a lack of signal in the latter two assay configurations). Furthermore, the generation of dose-response curves for the different configurations can enable shifts in potency as well as efficacy to be evaluated. Comparison of different signaling pathways can also reveal compounds exhibiting biased signaling as a consequence of heteromerization (Mustafa et al., 2012).

Finally, the amenability of Receptor-HIT for identifying heteromers or profiling/screening compounds is dependent upon the existence of a suitable “Protein C” for a particular receptor-receptor combination (**Figure 1**). For example, β-arrestin2 is a particularly good interacting partner for most GPCRs, and when it is not, G protein can often be utilized instead. It is also important not to make assumptions in terms of Protein C selection, as heteromerization can change the pharmacological profile in a manner that changes interactions with Protein C. This is illustrated by the findings with the α_1A_AR and the distinct β-arrestin2 recruitment profile when forming the α_1A_AR-CXCR2 heteromer (Mustafa et al., 2012).

## CONCLUDING REMARKS

 The formation of receptor complexes has a significant impact on cellular signaling. Receptor heteromers are of particular interest due to the unique biochemical profile they attain through heteromerization. Characterization of a heteromer’s biochemical fingerprint in heterologous expression systems is the first step to identifying the function of the heteromer in native tissue. Receptor-HIT is a novel cell-based approach for identifying and profiling heteromers that provides information on ligand-induced functional responses specific to the heteromer. The approach is highly versatile, allowing for simple and yet robust heteromer characterization. Utilizing a platform such as BRET, Receptor-HIT is a powerful tool that enables a deeper understanding of the molecular and physiological relevance of heteromers to be revealed.

## Conflict of Interest Statement

In addition to being Head of the Laboratory for Molecular Endocrinology – GPCRs,Western Australian Institute for Medical Research and Centre for Medical Research, The University of Western Australia, Associate Professor Kevin D. G. Pfleger is Chief Scientific Officer of Dimerix Bioscience, a spin-out company of The University of Western Australia that has been assigned the rights to the ‘Receptor-HIT’ technology. Associate Professor Kevin D. G. Pfleger has a minor shareholding in Dimerix.
